# 

**Published:** 1874-11

**Authors:** 


### CONTENTS:

ORIGINAL COMMUNICATIONS:
- ART. I. Sex and Contagion. By J. N. Hyde, M.D., Chicago........... 641
- ART. II. Koumiss, and its Use in Medicine. By O. C. DeWolf, M.D., Chicago........................... 658
- ART. III. Case of Injury to the Head. By G. J. Monroe, M.D., Leland, Ill. 665

REPORTS OF SOCIETIES:
- Chicago Society of Physicians and Surgeons............................ 667
- Chicago Medical Society............ 677

CLINICS:
- Rush Medical College................ 690

HOSPITALS:
- Cook County........................ 692
- St. Luke's.......................... 694

EDITORS' BOOK TABLE.................. 695

EDITORIAL............................ 700

CORRESPONDENCE....................... 701

OBITUARY............................. 702

MORTALITY STATISTICS................. 702

